# A Phase 2 Randomized Placebo-Controlled Adjuvant Trial of GI-4000, a Recombinant Yeast Expressing Mutated RAS Proteins in Patients with Resected Pancreas Cancer

**DOI:** 10.1089/pancan.2020.0021

**Published:** 2021-03-23

**Authors:** Peter Muscarella, Tanios Bekaii-Saab, Kristi McIntyre, Alexander Rosemurgy, Sharona B. Ross, Donald A. Richards, William E. Fisher, Patrick J. Flynn, Alicia Mattson, Claire Coeshott, Heinrich Roder, Joanna Roder, Frank E. Harrell, Allen Cohn, Timothy C. Rodell, David Apelian

**Affiliations:** ^1^ Department of Surgery, Ohio State University Comprehensive Cancer Center, Columbus, Ohio, USA.; ^2^ Mayo Clinic Cancer Center, Phoenix, Arizona, USA.; ^3^ Texas Oncology, US Oncology Research, Dallas, Texas, USA.; ^4^ Digestive Disorders Institute, AdventHealth Tampa, Tampa, Florida, USA.; ^5^ Texas Oncology, US Oncology Research, Tyler, Texas, USA.; ^6^ Baylor College of Medicine, Houston, Texas, USA.; ^7^ Minnesota Oncology, US Oncology Research, Minneapolis, Minnesota, USA.; ^8^ Smuggler Mountain Group (SMG, Inc.), Aspen, Colorado, USA.; ^9^ Biodesix, Inc., Boulder, Colorado, USA.; ^10^ Department of Biostatistics, Vanderbilt University School of Medicine, Nashville, Tennessee, USA.; ^11^ Rocky Mountain Cancer Center, Denver, Colorado, USA.

**Keywords:** *K-ras*, pancreas cancer, T cell response, clinical trial, immunotherapy

## Abstract

**Purpose:** GI-4000, a series of recombinant yeast expressing four different mutated RAS proteins, was evaluated in subjects with resected *ras*-mutated pancreas cancer.

**Methods:** Subjects (*n* = 176) received GI-4000 or placebo plus gemcitabine. Subjects' tumors were genotyped to identify which matched GI-4000 product to administer. Immune responses were measured by interferon-γ (IFNγ) ELISpot assay and by regulatory T cell (Treg) frequencies on treatment. Pretreatment plasma was retrospectively analyzed by matrix-assisted laser desorption/ionization-time-of-flight (MALDI-ToF) mass spectrometry for proteomic signatures predictive of GI-4000 responsiveness.

**Results:** GI-4000 was well tolerated, with comparable safety findings between treatment groups. The GI-4000 group showed a similar pattern of median recurrence-free and overall survival (OS) compared with placebo. For the prospectively defined and stratified R1 resection subgroup, there was a trend in 1 year OS (72% vs. 56%), an improvement in OS (523.5 vs. 443.5 days [hazard ratio (HR) = 1.06 [confidence interval (CI): 0.53–2.13], *p* = 0.872), and increased frequency of immune responders (40% vs. 8%; *p* = 0.062) for GI-4000 versus placebo and a 159-day improvement in OS for R1 GI-4000 immune responders versus placebo (*p* = 0.810). For R0 resection subjects, no increases in IFNγ responses in GI-4000–treated subjects were observed. A higher frequency of R0/R1 subjects with a reduction in Tregs (CD4^+^/CD45RA^+^/Foxp3^low^) was observed in GI-4000–treated subjects versus placebo (*p* = 0.033). A proteomic signature was identified that predicted response to GI-4000/gemcitabine regardless of resection status.

**Conclusion:** These results justify continued investigation of GI-4000 in studies stratified for likely responders or in combination with immune check-point inhibitors or other immunomodulators, which may provide optimal reactivation of antitumor immunity.

ClinicalTrials.gov Number: NCT00300950.

## Introduction

The *ras* oncogene and its RAS protein gene product contain the most common oncogene-related mutations in human cancer, with 90% of pancreas cancers harboring mutant RAS proteins.^1,2^ Mutations in the *ras* oncogene occur in conserved locations, specifically codons 12, 13, and 61,^3^ and the number of mutations that can occur is limited to a few predominant amino acid substitutions. RAS oncoproteins are theoretically ideal targets for cancer immunotherapy because aberrant signaling through RAS contributes to uncontrolled cell proliferation and tumorigenesis.

Cancer immunotherapies have employed many strategies to generate immune responses^4–10^ including cellular immunotherapies, which are showing much promise in advanced hematological cancers^11,12^ and immune check-point inhibitors, which have substantial activity in a number of solid tumors including melanoma,^13^ nonsmall cell lung cancer (NSCLC),^14^ and squamous cell head and neck cancers.^15,16^ In the study described here, our immunotherapeutic approach is based on the use of heat-killed recombinant *Saccharomyces cerevisiae* yeast as vectors, which are engineered to express target protein antigens. These yeast cells can activate dendritic cells and generate T cell cytotoxicity against target cells expressing viral and cancer antigens.^17–23^

The GI-4000 product series consists of four different yeast-based products that target the seven most common *ras* mutations at codons 12 and 61, all of which result in constitutive activation of RAS. Because of the central role for RAS activation in tumor proliferation, targeted destruction of cells harboring mutant RAS proteins could result in therapeutic benefit in human cancers. A phase 1 study in patients with pancreas and colorectal cancer indicated that GI-4000 was safe, well tolerated, and immunogenic.^24^ A phase 2b study in NSCLC patients also indicated that GI-4000 was well tolerated, and appeared to confer an overall survival (OS) benefit as compared with historical controls.^25^ Here we report the results of a randomized prospective trial of adjuvant gemcitabine versus gemcitabine plus GI-4000 in patients with resected pancreas cancer. The primary end-point was improvement in recurrence-free survival. Exploratory proteomic analysis was performed retrospectively to investigate signatures that might predict responsiveness to GI-4000.

## Methods

### Study oversight

The study protocol was approved by institutional review boards at each trial site. All patients gave written informed consent.

### Study design

This study was a randomized placebo-controlled double-blind adjuvant trial conducted at 27 investigational sites in the United States and 5 international sites in India and Bulgaria. After screening and informed consent, tumor tissue from surgical resection specimens was subjected to *ras* genomic sequencing. Subjects with mutations at either codon 12 or 61 positions represented in one of the GI-4000 products were eligible for study enrollment.

### Objectives

The primary objective of the study was to evaluate an improvement in recurrence-free survival with GI-4000 treatment. Key secondary objectives were to evaluate OS, safety, and immunogenicity.

### Variables

Demographic and baseline characteristics included age, gender, ethnic origin, time since diagnosis, tumor type, stage and grade, tumor biomarker levels, and *ras* gene mutations.

### Interventions

The study drug consisted of four different yeast-based products targeting the four most common *ras* mutations at codon 12 and the three most common *ras* mutations at codon 61 (GI-4014: G12V, Q61L, Q61R; GI-4015: G12C, Q61L, Q61R; GI-4016: G12D, Q61L, Q61R; GI-4020: G12R, Q61L, Q61H). Each subject received only the specific product containing the mutation identified in his or her tumor. The yeast strains were engineered to express the *K-ras* mutation insert sequences as previously described.^21^

The study population consisted of patients with resected pancreas cancer who had a product-related mutation in *ras* and an R0 or R1 resection by pancreaticoduodenectomy or pylorus-preserving pancreaticoduodenectomy procedure. An R0 resection was defined as no microscopic residual tumor at the resection margin. An R1 resection was defined as residual microscopic but not gross evidence of tumor at the resection margin. After enrollment, subjects were randomized in a 1:1 ratio to either GI-4000 or placebo, both combined with gemcitabine. It should be noted that adjuvant gemcitabine monotherapy was used as the control because at the time the trial was designed and recruited, neither recent data from ESPAC-4 nor data comparing gemcitabine with FOLFIRINOX were available, making gemcitabine monotherapy the standard of care. Randomization was prospectively stratified based on resection status (R0/R1). Subjects were dosed subcutaneously with 40 yeast units (YU; 1 YU = 10^7^ yeast cells) GI-4000 or with placebo (saline) for three weekly doses (0.5 mL/10 YU to each of four injection sites), starting 21 to 35 days after resection. Gemcitabine 1000 mg/m^2^ intravenous infusion was started on study Day 24. Monthly doses of GI-4000 or placebo were administered after initiation of gemcitabine to coincide with monthly chemotherapy holidays. Administration of gemcitabine proceeded until six monthly cycles were completed, intolerance occurred, study withdrawal, disease progression, or death. Administration of study drug proceeded until study withdrawal, disease recurrence, death, or completion of 60 months of therapy. A schematic of dosing for GI-4000 and gemcitabine is given in Table 1.

**Table 1.** Dosing Schedule and Immune Sampling Schedule

Day	1	8	15	24	31	38	44	52	59	66	72	80	87	94	100	108	115	122	128	136	143	150	156	164	171	178	184	212	240	
GEM				x	x	x		x	x	x		x	x	x		x	x	x		x	x	x		x	x	x				
GI-4000	x	x	x				x				x				x				x				x				x	x	x	Monthly
Immune sample	x		x	x			x	x							x	x											x			Quarterly Thereafter

Subjects were followed for up to 60 months after randomization and thereafter rolled into a long-term safety and outcomes protocol with an intended follow-up period of up to 15 years from treatment initiation.

### Tumor tissue sequencing

Cellular genomic DNA was extracted from biopsy material and analyzed to identify *ras* mutations as previously described.^24^

### Immunology analyses

Analyses were performed on samples blinded to treatment. Peripheral blood mononuclear cells (PBMCs) were collected and cryopreserved until use. Testing was performed on samples from subjects enrolled at sites in the United States only. Interferon-γ (IFNγ) ELISpot assays were performed as previously described.^25^ Immunophenotyping by flow cytometry evaluated the frequency of regulatory T cell (Treg) fractions,^26^ using PBMCs from baseline and Day 15 or Day 24 time points.

### Exploratory proteomic analysis

Baseline plasma samples were retrospectively analyzed by matrix-assisted laser desorption/ionization (MALDI) time-of-flight (ToF) mass spectrometry.

### Statistical methods

A Bayesian statistical approach was used to analyze efficacy on a quarterly basis using time to recurrence as the primary efficacy end-point and time to mortality as a key secondary efficacy end-point. Enrollment was expanded beyond the originally planned 100 patients based on the probability of improved efficacy for time to recurrence of <0.95 and >0.70 (this range of probabilities represents a strong trend, i.e., not yet definitive) and if an estimate of increased time to recurrence and mortality exceeded 2 months during enrollment. The efficacy analysis supported sample size expansion up to 176 patients overall, with 39 patients in the R1 subgroup and 137 patients in the R0 subgroup. Enrollment was permitted to continue until the prespecified limits were met. Once the boundaries were exceeded, the study ceased to accrue new patients.

## Results

### Participants

**FIG. 1.**
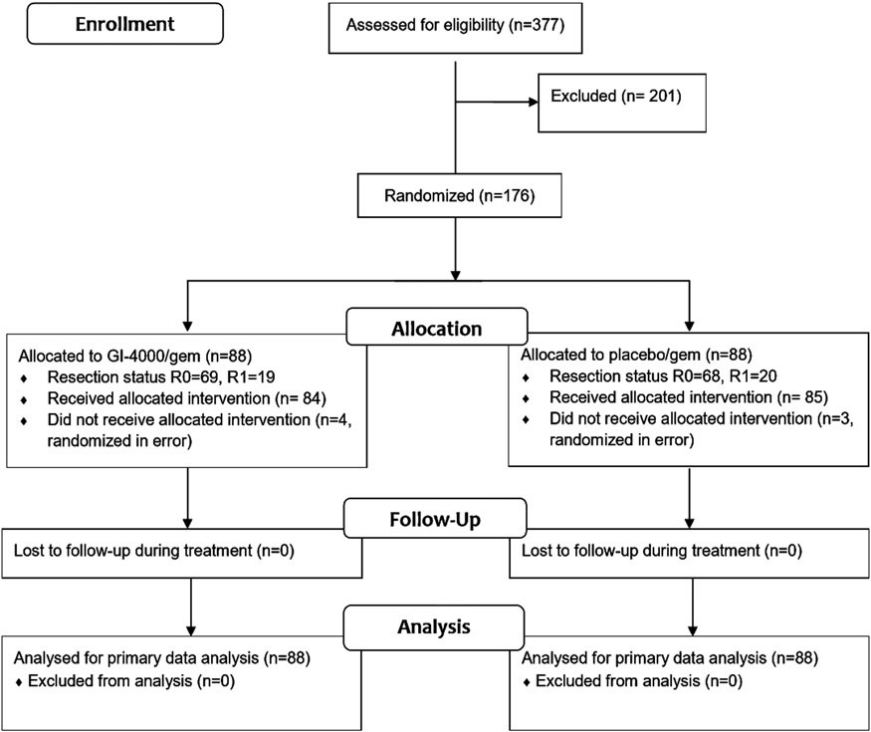
Study disposition. n, number of subjects.

Study disposition is shown in Figure 1. A total of 377 R0/R1 subjects were screened and 176 subjects were subsequently randomized to receive GI-4000 + gemcitabine (88 subjects), or placebo + gemcitabine (88 subjects) between June 5, 2006, and April 30, 2010. These subjects comprised the intent-to-treat (ITT) population. The safety population consisted of a total of 169 subjects who received at least one dose of study drug: 84 subjects received GI-4000 and 85 subjects received placebo. The primary reasons for screened subjects failing to enroll included either the lack of a *K-ras* mutation in their tumor or the presence of a mutation not represented in the GI-4000 products.

Unless otherwise stated, analyses are for the ITT population who underwent R0/R1 resection. The most common reason for study discontinuation in both treatment groups was death (111 subjects, 63.1% of the ITT population).

Table 2 summarizes the baseline demographic and disease characteristics. The mean age was 62.1 years and the majority of the treated subjects were white (80.7%) and men (58.5%). The *ras* mutations present in tumors were similar between treatment groups and most subjects with R0/R1 resection in both treatment groups had either a G12V (44.3%) or a G12D (43.2%) mutation. Most subjects in both groups had a baseline Eastern Cooperative Oncology Group (ECOG) Performance Status of either Grade 1 (59.1%) or Grade 0 (25.0%).

**Table 2.** Summary of Baseline Demographic and Disease Characteristics for All Enrolled Subjects

	n (%)		
Parameter	GI-4000 + gemcitabine	Placebo + gemcitabine	Total
Age (years)	63.4	60.8	62.1
Gender			
Women	34 (38.6%)	39 (44.3%)	73 (41.5%)
Men	54 (61.4%)	49 (55.7%)	103 (58.5%)
Race			
White	71 (80.7%)	71 (80.7%)	142 (80.7%)
African American	7 (8.0%)	7 (8.0%)	14 (8.0%)
Asian	4 (4.5%)	4 (4.5%)	8 (4.5%)
Hispanic	6 (6.8%)	5 (5.7%)	11 (6.3%)
Other	0	1 (1.1%)	1 (0.6%)
ECOG performance status			
Grade 0	22 (25.0%)	22 (25.0%)	44 (25.0%)
Grade 1	52 (59.1%)	52 (59.1%)	104 (59.1%)
Grade 2	8 (9.1%)	6 (6.8%)	14 (8.0%)
Grade 3	0 (0.0%)	1 (1.1%)	1 (0.6%)
Not reported	6 (6.8%)	7 (8.0%)	13 (7.4%)
*ras* mutation			
G12V^a^	41 (46.6%)	37 (42.0%)	78 (44.3%)
G12C	3 (3.4%)	0 (0.0%)	3 (1.7%)
G12D	35 (39.8%)	41 (46.6%)	76 (43.2%)
G12R	7 (8.0%)	8 (9.1%)	15 (8.5%)
Q61H	2 (2.3%)	2 (2.3%)	4 (2.3%)
Primary tumor			
T1	7 (8.0%)	9 (10.2%)	16 (9.1%)
T2	8 (9.1%)	10 (11.4%)	18 (10.2%)
T3	70 (79.5%)	68 (77.3%)	138 (78.4%)
T4	3 (3.4%)	0 (0.0%)	3 (1.7%)
Not reported	0 (0.0%)	1 (1.1%)	1 (0.6%)
Regional lymph node status			
N0	25 (28.4%)	20 (22.7%)	45 (25.6%)
N1	34 (38.6%)	36 (40.9%)	70 (39.8%)
N1a	6 (6.8%)	9 (10.2%)	15 (8.5%)
N1b	23 (26.1%)	22 (25.0%)	45 (25.6%)
Not reported	0 (0.0%)	1 (1.1%)	1 (0.6%)
CA 19–9 (U/mL) postoperative			
Number of subjects	84	84	168
Median	24.9	16.1	18.8
Normal	59 (67.0%)	63 (71.6%)	122 (69.3%)
Abnormal^b^	25 (28.4%)	21 (23.9%)	46 (26.1%)
Not reported	4 (4.5%)	4 (4.5%)	8 (4.5%)

^a^ G, glycine; C, cysteine; D, aspartic acid; R, arginine; Q, glutamine; H, histidine; L, leucine; V, valine.

^b^ CA 19-9 values >35 U/mL were classified as abnormal.

ECOG, Eastern Cooperative Oncology Group.

Most primary tumors were stage pT3 (138 subjects, 78.4%) and there were no significant differences here between the GI-4000 and placebo cohorts (79.5% vs. 77.3%, respectively). Three subjects had T4 primary lesions and all were randomized to the GI-4000 group. The status of regional lymph node involvement was comparable between treatment groups. A higher percentage of subjects in the placebo group than in the GI-4000 group had metastasis in a single regional lymph node (10.2% vs. 6.8%, respectively), whereas metastasis in multiple regional lymph nodes occurred in a similar percentage of subjects in the GI-4000 and placebo groups (26.1% vs. 25.0%, respectively).

### Efficacy

**FIG. 2.**
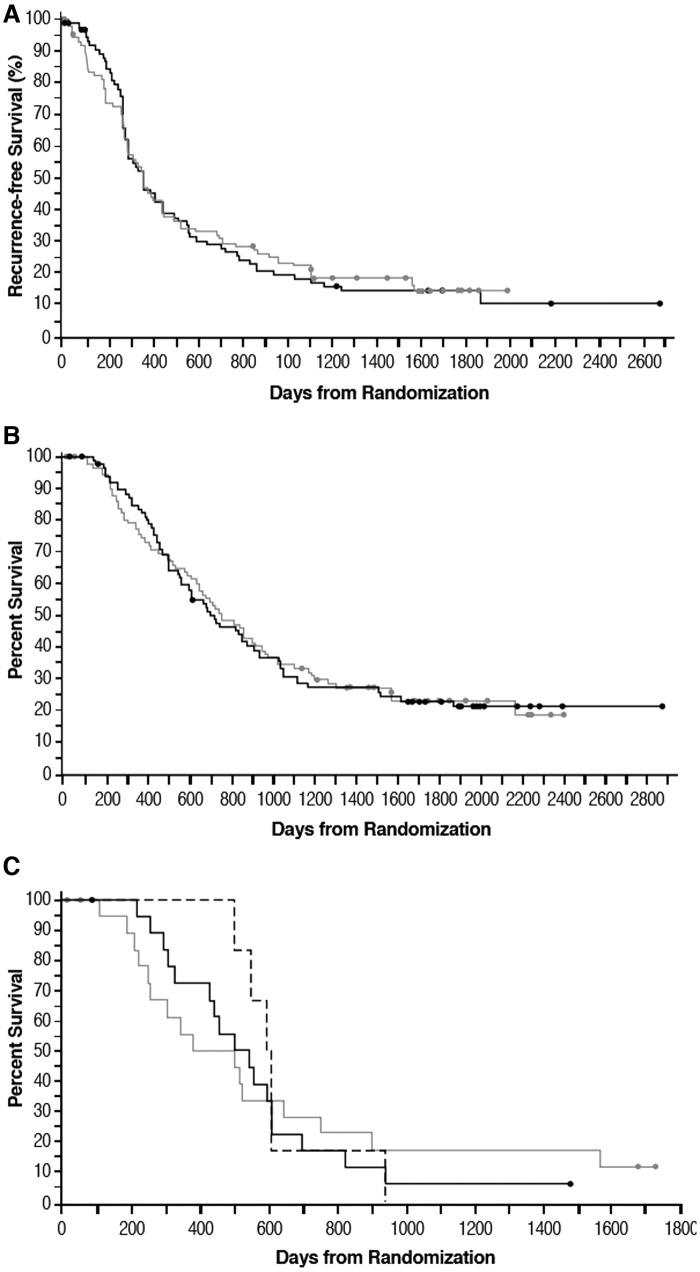
Recurrence-free survival **(A)** and OS **(B, C)** based on Kaplan–Meier estimates of all enrolled subjects (ITT population) **(A, B)** or ITT subjects with R1 resection **(C)** from date of randomization. Black line, GI-4000; gray line, placebo; dashed black line, GI-4000 treated R1 IFNγ ELISpot responders. Circles, censored data. A single subject in the R1 placebo group was an IFNγ ELISpot responder and had an OS of 382 days. IFNγ, interferon-γ; ITT, intent-to-treat; OS, overall survival.

The median time from randomization to recurrence was similar for the GI-4000 and placebo groups at 354 and 357 days, respectively (hazard ratio [HR] = 1.01 [95% confidence intervals (CIs): 0.73–1.41], *p* = 0.936). The percentage of subjects free of recurrence in the GI-4000 group was similar to that of the placebo group (18.2% vs. 17.0%, respectively). The median time from randomization to death was also similar for the GI-4000 and placebo groups: 698 versus 751 days, respectively (HR = 1.01 [CI: 0.72–1.42], *p* = 0.956). Kaplan–Meier estimates of the duration of radiological recurrence-free survival and of OS from randomization show comparable patterns for both treatment groups (Fig. 2).

For the prospectively defined and stratified R1 subgroup, there was a nonsignificant trend for improved OS with GI-4000 treatment, with an advantage in 1-year survival for GI-4000 versus placebo (72.2% vs. 55.6%, respectively), and an improvement in median OS of 80 days for GI-4000 versus placebo (523.5 vs. 443.5 days, HR = 1.06 [CI: 0.53–2.13], *p* = 0.872).

### Safety

The side effect and safety profiles of subjects receiving GI-4000/gemcitabine were similar to those of subjects receiving placebo/gemcitabine. Table 3 summarizes treatment emergent adverse events (TEAEs) occurring in at least 5% of ITT subjects and occurring in ≥30 of all subjects. The most frequent TEAEs were fatigue (55.1%), nausea (51.7%), anemia (42.6%), diarrhea (42.6%), and neutropenia (41.5%). Overall, the frequencies of adverse events were comparable between treatment groups and consistent with events expected in the population being studied. The TEAE that occurred with a notably higher incidence in the GI-4000 group than the placebo group was injection site pain (25.0% vs. 3.4%). A notably higher incidence in the placebo group occurred with the TEAE of depression (11.4% GI-4000 vs. 23.9% placebo).

**Table 3.** Summary of Treatment Emergent Adverse Events for ≥5% of all Subjects and ≥30 of All Subjects

MedDRA System Organ Class Preferred term	GI-4000 + Gemcitabine N = 88	Placebo + Gemcitabine N = 88	Total N = 176
Blood and lymphatic system disorders, *n* (%)			
Anemia	36 (40.9)	39 (44.3)	75 (42.6)
Neutropenia	37 (42.0)	36 (40.9)	73 (41.5)
Thrombocytopenia	15 (17.0)	17 (19.3)	32 (18.2)
Gastrointestinal disorders, *n* (%)			
Abdominal pain	33 (37.5)	32 36.4)	65 (36.9)
Constipation	26 (29.5)	27 (30.7)	53 (30.1)
Diarrhea	31 (35.2)	44 (50.0)	75 (42.6)
Nausea	46 (52.3)	45 (51.1)	91 (51.7)
Vomiting	25 (28.4)	24 (27.3)	49 (27.8)
General disorders and administration site conditions, *n* (%)			
Fatigue	47 (53.4)	50 (56.8)	97 (55.1)
Edema, peripheral	29 (33.0)	26 (29.5)	55 (31.3)
Pyrexia	25 (28.4)	30 (34.1)	55 (31.3)
Injection site reactions^a^	40 (45.5)	8 (9.1)	48 (27.3)
Metabolism and nutrition disorders, *n* (%)			
Anorexia	16 (18.2)	16 (18.2)	32 (18.2)
Musculoskeletal and connective tissue disorders, *n* (%)			
Back pain	22 (25.0)	19 (21.6)	41 (23.3)
Nervous system disorders, *n* (%)			
Dizziness	16 (18.2)	17 (19.3)	33 (18.8)
Headache	20 (22.7)	19 (21.6)	39 (22.2)
Psychiatric disorders, *n* (%)			
Depression	10 (11.4)	21 (23.9)	31 (17.6)
Insomnia	22 (25.0)	15 (17.0)	37 (21.0)

^a^ includes injection site erythema, induration, and pain.

### Immunogenicity

#### IFNγ ELISpot response

There was no difference in the frequency of ELISpot responders between the treatment groups with 22 of 67 (32.8%) subjects in the GI-4000 group versus 23 of 62 (37.1%) subjects in the placebo-treated group (Table 4). However, there was a nonsignificant increase in frequency of ELISpot responders in the R1 subgroup treated with GI-4000, with 6 of 15 subjects tested for GI-4000 versus 1 of 12 subjects tested for placebo (40.0% vs. 8.3%; *p* = 0.062). In addition, there was an improvement in median OS of 159 days for the R1 GI-4000 ELISpot immune responders versus all placebo-treated subjects (*p* = 0.810). For the R0 subgroup, there were comparable categorical ELISpot responses in both treatment groups with 16 of 52 (30.8%) responders in the GI-4000 group compared with 22 of 50 (44.0%) responders in the placebo group.

**Table 4.** Summary of Immune Responders

Treatment	IFNγ ELISpot response						Naïve Treg fraction reduction					
	R1		R0		R0/R1		R1		R0		R0/R1	
	Total subjects tested	Responders (%)	Total subjects tested	Responders(%)	Total subjects tested	Responders (%)	Total subjects tested	Responders (%)	Total subjects tested	Responders (%)	Total subjects tested	Responders (%)
GI-4000	15	6 (40.0)^a^	52	16 (30.8)	67	22 (32.8)	9	3 (33.3)	42	11 (26.2)	51	14 (27.4)^b^
Placebo	12	1 (8.3)	50	22 (44.0)	62	23 (37.1)	7	1 (14.3)	34	3 (8.8)	41	4 (9.7)

An increase or decrease in Tregs on treatment was defined as at least a twofold change in frequency from baseline.

^a^ *p* = 0.062, Pearson chi-square test, for GI-4000 versus placebo groups.

^b^ *p* = 0.033, Pearson chi-square test, for GI-4000 versus placebo groups.

IFNγ, interferon-γ.

#### Treg phenotyping

There was a threefold greater frequency for a decrease in naive Tregs (CD4^+^CD45RA^+^Foxp3^low^) in the GI-4000 group, with 14 of 51 subjects (27.4%) showing a twofold or greater decrease compared with 4 of 41 (9.7%) placebo subjects (*p* = 0.033) (Table 4).

### Proteomic analysis

Baseline plasma samples (44 in the GI-4000 group and 46 in the placebo group) were retrospectively analyzed by exploratory MALDI-ToF mass spectrometry using previously described methods.^27^ A classifier, BDX-001, was created using a strongly regularized logistic regression combination of five nearest neighbor classifiers composed of single or pairs of 100 mass spectral features (Supplementary Data). The training set for the classifier consisted of 23 samples from GI-4000–treated patients. Classifier performance was assessed on the remaining 21 samples for the GI-4000 group and all 46 placebo group samples.

**FIG. 3.**
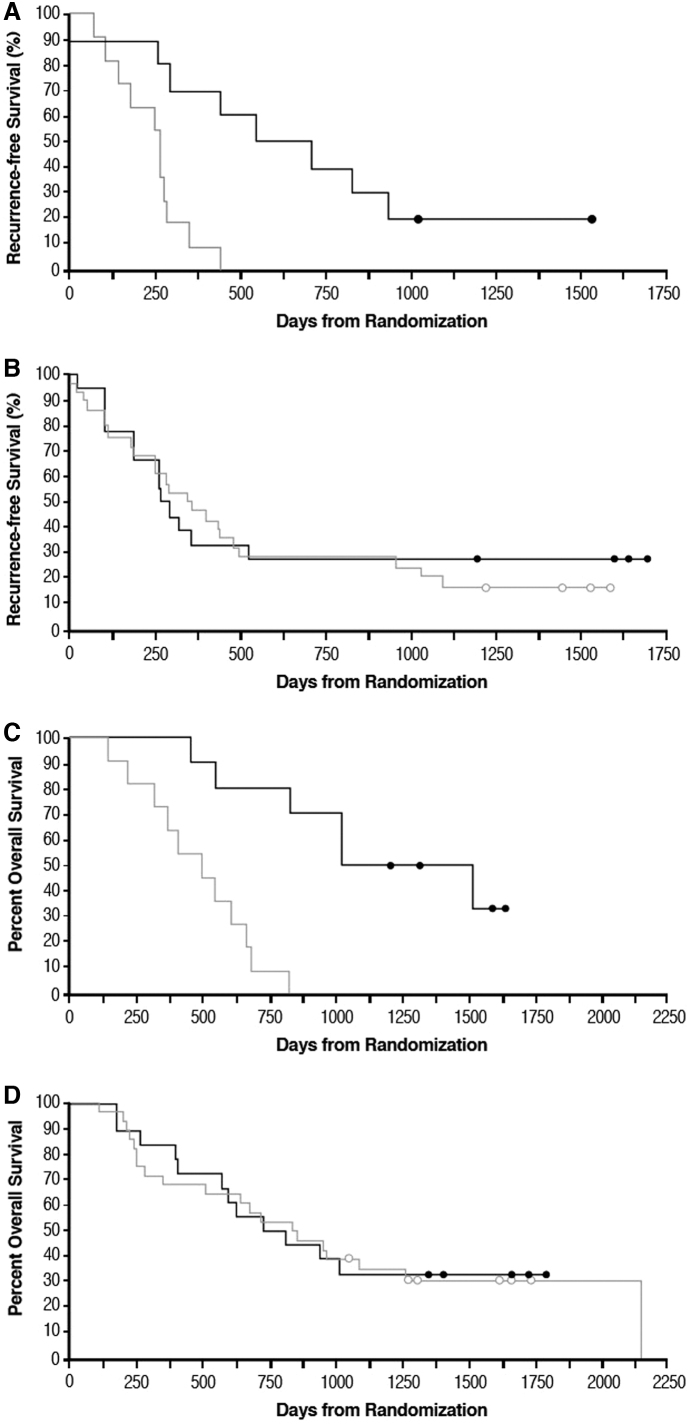

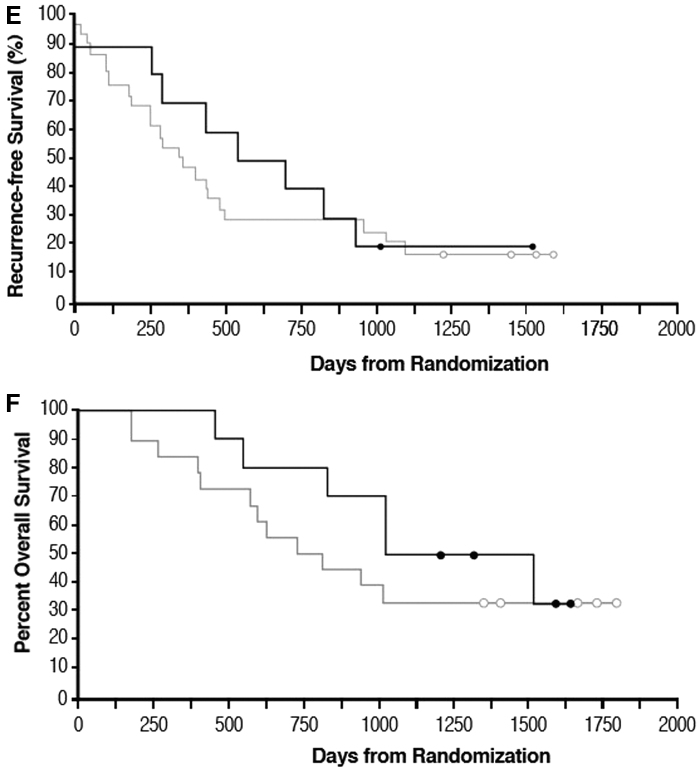
Recurrence-free survival **(A, B, E)** and OS **(C, D, F)** based on Kaplan–Meier estimates for subjects treated with GI-4000 or placebo and analyzed for proteomic signature. Baseline samples from 44 subjects in the GI-4000–treated group and 46 subjects in the placebo-treated group were available to investigate the proteomic signature. Samples from GI-4000–treated subjects were further subdivided into training (*n* = 23) and test (*n* = 21) sets and all samples not used in the classifier training set were designated BDX-001+ or BDX-001−. Circles, censored data. **(A)** Recurrence-free survival for GI-4000–treated subjects with (*n* = 10, black line)/without (*n* = 11, gray line) BDX-001 signature. **(B)** Recurrence-free survival for placebo-treated subjects with (*n* = 18, black line)/without (*n* = 28, gray line) BDX-001 signature. **(C)** OS for GI-4000–treated subjects with (*n* = 10, black line)/without (*n* = 11, gray line) BDX-001 signature. **(D)** OS for placebo-treated subjects with (*n* = 18, black line)/without (*n* = 28, gray line) BDX-001 signature. **(E)** Recurrence-free survival for BDX-001+ GI-4000 (*n* = 10, black line) and placebo-treated subjects (*n* = 18, gray line). **(F)** OS for BDX-001+ GI-4000 (*n* = 10, black line) and placebo-treated subjects (*n* = 18, gray line).

The classifier divided subjects into two classes, BDX-001+ and BDX-001−, with, respectively, better and worse outcomes when treated with GI-4000: treated subjects classified as BDX-001+ had a 12.0 month improvement in recurrence-free survival compared with GI-4000–treated subjects classified as BDX-001− (HR = 0.30 [CI: 0.07–0.49], *p* = 0.002, Fig. 3A). In contrast, there was no improvement in recurrence-free survival between BDX-001+ and BDX-001− placebo subjects (unfavorable 2.4 months difference, HR = 1.11 [CI: 0.57–2.18], *p* = 0.754, Fig. 3B). When used to evaluate OS, the proteomic classifier also predicted better and worse survival for subjects in the GI-4000 group (25.4 months improvement BDX-001+ vs. BDX-001−, HR = 0.21 [CI: 0.04–0.31], *p* < 0.001, Fig. 3C) but not the placebo group (HR = 1.03 [CI: 0.50–2.10], *p* = 0.944, Fig. 3D). BDX-001+ subjects treated with GI-4000 had improved recurrence-free survival and OS compared with BDX-001+ placebo subjects with an 11.5 months improvement in recurrence-free survival: 20.7 months versus 9.2 months (HR = 0.80 [CI: 0.34–1.91], *p* = 0.623) and a nonsignificant 16.4 months improvement in median OS (41.9 months for GI-4000 vs. 25.5 months for placebo, HR = 0.65 [CI: 0.26–1.67], *p* = 0.384) (Fig. 3E and F, respectively).

When this proteomic classifier was applied to only R0 subjects from both treatment groups, an advantage in median recurrence-free survival of 13.7 months was observed for GI-4000 compared with placebo (23.2 vs. 9.5 months, HR = 0.68 [CI: 0.27–1.73], *p* = 0.426) and an advantage in median OS of 25.7 months was observed (49.9 months for GI-4000 vs. 24.2 months for placebo, HR = 0.46 [CI: 0.18–1.25], *p* = 0.135) for BDX-001+ subjects.

## Discussion

This phase 2 study was a randomized double-blind placebo-controlled multicenter trial comparing GI-4000 plus gemcitabine with placebo plus gemcitabine in subjects with resected *ras*-mutated pancreas cancer. Subjects were prospectively stratified based on their resection status (R0/R1). Since the majority of subjects in the trial were in the R0 subgroup (137/176; 78%), the overall findings in the study (R0 and R1 subjects) mirror those of the R0 subgroup analyses, including recurrence-free survival, OS, and mortality. To appreciate the potential differences observed in these subgroups, data have, therefore, been also analyzed separately.

The R1 subgroup showed an increase in subjects with T cell ELISpot responses after GI-4000 treatment compared with placebo treatment, and nonsignificant advantages in 1-year OS for GI-4000 versus placebo and an improvement in median OS of ∼3 months for GI-4000. Furthermore, there was a nonsignificant >5 months improvement in median OS for the R1 GI-4000 ELISpot immune responders versus placebo, indicating a potential mechanism-based improvement in survival for the R1 subgroup. In contrast, the R0 group showed comparable ELISpot responses in both treatment groups, indicating that there appears to be a greater tendency for background tumor-specific immune responses in R0 subjects than in R1 subjects.

Tregs are known to be overexpressed in pancreas cancer^28^ and poor prognosis is associated with the presence of Tregs in the periphery or in the tumor microenvironment.^29–32^ In this study, GI-4000 treatment rapidly decreased the naive Treg subpopulation. This decrease could be a potential mechanism of action of GI-4000 that contributes to effects on recurrence and survival. Since the GI-4000 vector is yeast based, it may reduce the number and function of Tregs through reciprocal activation of the Th17 T cell pathway.^33–35^

The improved ELISpot responses seen in the GI-4000–treated R1 subgroup, together with a trend in improved survival for all GI-4000–treated R1 subjects, suggest residual antigen may be required for optimal response. Reduction in Tregs by GI-4000 may act preferentially for R1 subjects by allowing effector T cells generated by GI-4000 to infiltrate the tumor where the presence of RAS antigen within the residual tumor margins could further drive the effector T cell response. The absence of an intact tumor in R0 subjects may, therefore, not reveal these dual benefits of GI-4000 treatment. Because of the small sample size in the R1 group, if the survival benefit in this group is real, a substantially larger trial would be required to confirm it.

Improved survival with GI-4000 treatment was retrospectively defined by a proteomic signature. The difference in time to recurrence between BDX-001+ and BDX-001− subjects treated with GI-4000 was statistically significant and did not depend on resection status. These survival trends indicate that this proteomic signature predicted late recurrence in the GI-4000–treated subjects, but not placebo subjects, and could potentially be used as an enrichment bioassay to improve observed treatment effects in future clinical trials, as demonstrated for a predictive classifier in responses of NSCLC patients to erlotinib and chemotherapy.^36^

GI-4000 was shown to be well tolerated, with safety findings comparable between the two groups and with no differences noted for R0 and R1 subjects. Overall, the GI-4000 group showed a similar pattern of recurrence-free survival and OS compared with the placebo group.

The *K-ras* mutation G12C has recently been exploited to design small molecule inhibitors that show promise for NSCLC treatment.^37^ However, as illustrated here, mutations in *K-ras* in pancreatic cancer are predominantly G12V and G12D; there was only a single subject with a G12C *K-ras* mutation in our study. Therefore, small molecule inhibitors for deployment in pancreatic cancer are still being sought. It may be beneficial to combine GI-4000 with cellular immunotherapies such as chimeric antigen receptor T cells or tumor-infiltrating lymphocytes^38,39^ in pancreatic cancer as GI-4000 may synergize to provide antigen-specific stimuli for the infused T cells. In addition, use of check-point inhibitors to block T cell death pathways may provide optimal reactivation of antitumor immunity in combination with GI-4000. Clinical trials are currently in progress or planned in a number of tumor types combining GI-4000 with other immune therapies and chemotherapies.^40^ As previously mentioned, it should also be noted that, given the promise of new regimens using capecitabine and FOLFIRINOX,^41,42^ gemcitabine can probably no longer be considered standard of care in pancreatic cancer patients and any future studies will almost certainly employ a different control arm.

## Conclusion

Given the current promise of immunotherapy and interest in strategies to target cancer patients likely to respond to treatments, we believe continued investigation of GI-4000 is warranted, with further prospective studies stratified for likely responders. Combination with immune check-point inhibitors or other immunomodulators may also be beneficial as this may provide optimal reactivation of antitumor immunity.

## Supplementary Material

Supplemental data

## Notes

**Cite this article as:** Muscarella P, Bekaii-Saab T, McIntyre K, Rosemurgy A, Ross SB, Richards DA, Fisher WE, Flynn PJ, Mattson A, Coeshott C, Roder H, Roder J, Harrell FE, Jr., Cohn A, Rodell TC, Apelian D (2021) A phase 2 randomized placebo-controlled adjuvant trial of GI-4000, a recombinant yeast expressing mutated RAS proteins in patients with resected pancreas cancer, *Journal of Pancreatic Cancer* 7:1, 8–19, DOI: 10.1089/pancan.2020.0021.

## Abbreviations Used

CI: confidence interval
ECOG: Eastern Cooperative Oncology Group
FU: follow-up
HR: hazard ratio
IFNγ: interferon-γ
ITT: intent-to-treat
MALDI-ToF: matrix-assisted laser desorption/ionization-time-of-flight
*N, n*: number of subjects
NSCLC: nonsmall cell lung cancer
OS: overall survival
PBMCs: peripheral blood mononuclear cells
TEAEs: treatment emergent adverse events
Treg: regulatory T cell
YU: yeast units
