# Supplementary material for: A Phase 2 Randomized Placebo-Controlled Adjuvant Trial of GI-4000, a Recombinant Yeast Expressing Mutated RAS Proteins in Patients with Resected Pancreas Cancer
Source: J Pancreat Cancer. 2021 Mar 23;7(1):8–19. doi: 10.1089/pancan.2020.0021 (PMC7997807; doi:10.1089/pancan.2020.0021)
Supplement: Supplemental data [file Supp_Table1.docx]

The BDX-001 Proteomic Signature

BDX-001 classification is generated based on the value of 100 mass spectral features. A mass spectral feature is defined as a region in the mass spectrum. The value of each mass spectral feature for a sample is the intensity of the processed mass spectrum generated from that sample integrated across the feature. (Mass spectra require processing prior to feature value determination to make them comparable between samples.) The locations of the 100 mass spectral features used in BDX-001 classification are given in Table S1.

Table S1: Mass spectral features used in classification (m/Z in Daltons)

| 3108 | 4962 | 8762 | 11883 |
| --- | --- | --- | --- |
| 3130 | 4997 | 8894 | 11901 |
| 3217 | 5020 | 8912 | 12837 |
| 3236 | 5066 | 8992 | 12856 |
| 3246 | 5104 | 9566 | 12955 |
| 3266 | 5136 | 9664 | 12976 |
| 3368 | 5193 | 9707 | 13062 |
| 3428 | 5391 | 9750 | 13145 |
| 3463 | 5571 | 9863 | 13264 |
| 3723 | 5718 | 10000 | 13306 |
| 3841 | 5734 | 10071 | 13706 |
| 3893 | 5762 | 10091 | 13735 |
| 3935 | 5776 | 10201 | 13782 |
| 4135 | 5841 | 11380 | 13901 |
| 4186 | 5862 | 11402 | 14031 |
| 4207 | 5907 | 11432 | 14112 |
| 4289 | 6171 | 11466 | 14138 |
| 4444 | 6772 | 11488 | 14281 |
| 4458 | 6893 | 11521 | 22988 |
| 4469 | 6982 | 11544 | 23021 |
| 4567 | 7041 | 11620 | 23221 |
| 4624 | 7240 | 11676 | 28038 |
| 4686 | 7385 | 11699 | 28232 |
| 4789 | 7690 | 11723 | 28439 |
| 4855 | 8357 | 11744 | 28805 |

# The BDX-001 classification algorithm is a strongly dropout-regularized logistic regression combination of 5-nearest neighbor (5NN) classifiers formed from individual features and pairs of features. The output of the regression is converted to a binary result, BDX-001+ or BDX-001- depending on whether it is greater than or less than a fixed cutoff of 0.5. More details on this classification method can be found in Roder et al. *A dropout-regularized classifier development approach optimized for precision medicine test discovery from omics data*. BMC Bioinformatics. 2019. 20(1):325. Briefly, the algorithm is a logistic regression of the outputs of 5NN classifiers constructed using single features or pairs of features (from Table S1). The large number of 5NN classifiers using pairs of features were filtered to reject those showing no sign of utility for stratification of patient outcome when treated with GI-4000, using only data from the training set. The weights in the regression were determined in training using extreme dropout (Srivastava et al. *Dropout: A simple way to prevent neural networks from overfitting*. Journal of Machine Learning Research 2014. 15:1929-58), which retains only 0.1% of the 5NNs in each of 10,000 dropout iterations to minimize the chance of overfitting given the small size of the training set.
